# Supplementary material for: A network dynamical simulation model for the study of antibiotic resistance in microbial communities
Source: Gut Microbes. 2026 Sep 19;18(1):2734703. doi: 10.1080/19490976.2026.2734703 (PMC13596919; doi:10.1080/19490976.2026.2734703)
Supplement: Supplementary file 1 [file KGMI_A_2734703_SM9053.zip › supplementary_file_1/Figures/all_100nodes_ab60_resistance_timeseries.pdf]

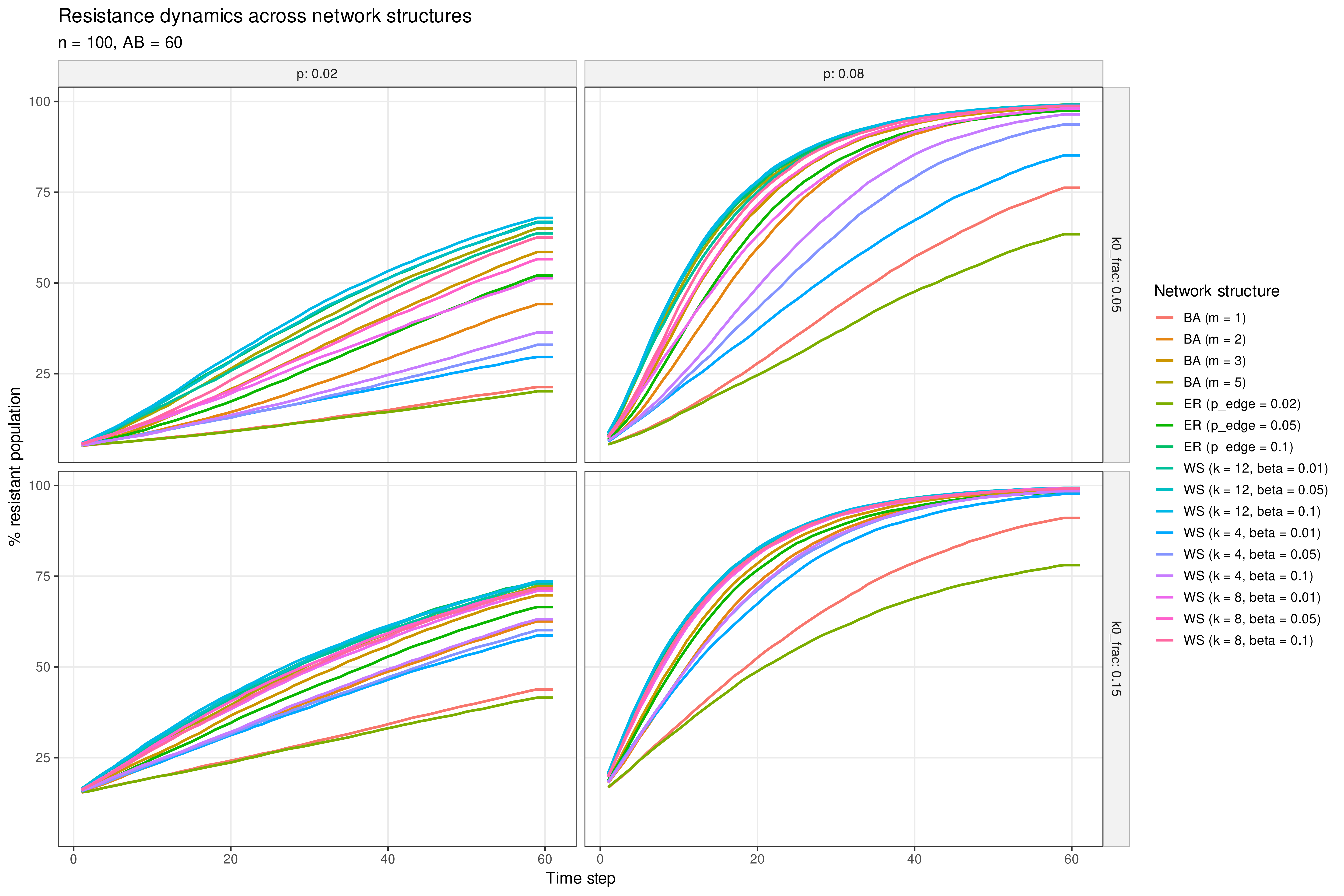

Resistance dynamics across network structures
n = 100, AB = 60
p: 0.02
p: 0.08
k0_frac: 0.05
k0_frac: 0.15
% resistant population
Time step
100
75
50
25
0
20
40
60
Network structure
BA (m = 1)
BA (m = 2)
BA (m = 3)
BA (m = 5)
ER (p_edge = 0.02)
ER (p_edge = 0.05)
ER (p_edge = 0.1)
WS (k = 12, beta = 0.01)
WS (k = 12, beta = 0.05)
WS (k = 12, beta = 0.1)
WS (k = 4, beta = 0.01)
WS (k = 4, beta = 0.05)
WS (k = 4, beta = 0.1)
WS (k = 8, beta = 0.01)
WS (k = 8, beta = 0.05)
WS (k = 8, beta = 0.1)
